# Finding Holes: Pathologist-Level Performance Using AI for Cribriform Morphology Detection in Prostate Cancer

**DOI:** 10.1016/j.euros.2026.03.016

**Published:** 2026-04-07

**Authors:** Kelvin Szolnoky, Anders Blilie, Nita Mulliqi, Toyonori Tsuzuki, Hemamali Samaratunga, Matteo Titus, Xiaoyi Ji, Sol Erika Boman, Einar Gudlaugsson, Svein Reidar Kjosavik, José Asenjo, Marcello Gambacorta, Paolo Libretti, Marcin Braun, Radzisław Kordek, Roman Łowicki, Brett Delahunt, Kenneth A. Iczkowski, Theo van der Kwast, Geert J.L.H. van Leenders, Katia R.M. Leite, Chin-Chen Pan, Emiel Adrianus Maria Janssen, Martin Eklund, Lars Egevad, Kimmo Kartasalo

**Affiliations:** a Department of Medical Epidemiology and Biostatistics, Karolinska Institutet, Stockholm, Sweden; b Department of Pathology, Stavanger University Hospital, Stavanger, Norway; c Faculty of Health Sciences, University of Stavanger, Stavanger, Norway; d Department of Surgical Pathology, School of Medicine, Aichi Medical University, Nagoya, Japan; e Aquesta Uropathology and University of Queensland, Brisbane, Queensland, Australia; f Department of Molecular Medicine and Surgery, Karolinska Institutet, Stockholm, Sweden; g The General Practice and Care Coordination Research Group, Stavanger University Hospital, Stavanger, Norway; h Department of Global Public Health and Primary Care, Faculty of Medicine, University of Bergen, Bergen, Norway; i Department of Pathology, SYNLAB, Madrid, Spain; j Department of Pathology, SYNLAB, Brescia, Italy; k Department of Pathology, Chair of Oncology, Medical University of Lodz, Lodz, Poland; l 1st Department of Urology, Medical University of Lodz, Lodz, Poland; m Malaghan Institute of Medical Research, Wellington, New Zealand; n Department of Oncology and Pathology, Karolinska Institutet, Stockholm, Sweden; o Department of Pathology and Laboratory Medicine, University of California - Davis Health, Sacramento, CA, USA; p Laboratory Medicine Program and Princess Margaret Cancer Center, University Health Network, University of Toronto, Toronto, ON, Canada; q Department of Pathology, Erasmus MC, University Medical Center, Rotterdam, the Netherlands; r Department of Urology, Laboratory of Medical Research, University of São Paulo Medical School, São Paulo, Brazil; s Department of Pathology and Laboratory Medicine, Taipei Veterans General Hospital, Taipei, Taiwan; t Department of Chemistry, Bioscience and Environmental Engineering, University of Stavanger, Stavanger, Norway; u Institute for Biomedicine and Glycomics, Griffith University, Brisbane, Queensland, Australia; v Department of Medical Epidemiology and Biostatistics, SciLifeLab, Karolinska Institutet, Stockholm, Sweden

**Keywords:** Artificial intelligence, Cribriform, Prostate cancer

## Abstract

**Background:**

Cribriform morphology in prostate cancer is a histological feature that indicates poor prognosis and contraindicates active surveillance. However, it remains underreported and subject to significant interobserver variability among pathologists.

**Objective:**

We aimed to develop and validate an artificial intelligence (AI)-based system to improve cribriform pattern detection.

**Design, setting, and participants:**

We created a deep learning model using an EfficientNetV2-S encoder with multiple instance learning for end-to-end whole-slide classification. The model was trained on 640 digitised prostate core needle biopsies from 430 patients, collected across three cohorts. It was validated internally (261 slides from 171 patients) and externally (266 slides, 104 patients from three independent cohorts). Internal validation cohorts included laboratories or scanners from the development set, while external cohorts used completely independent instruments and laboratories. Annotations were provided by three expert uropathologists with known high concordance.

**Outcome measurements and statistical analysis:**

We assessed model performance using the area under the receiver operating characteristic curve (AUC) and Cohen’s κ with 95% confidence intervals calculated through bootstrapping. Additionally, we conducted an inter-rater analysis and compared the model’s performance against nine expert uropathologists on 88 slides from the internal validation cohort.

**Results and limitations:**

The model showed strong internal validation performance (AUC: 0.97, 95% CI: 0.95, 0.99; Cohen’s κ: 0.81, 95% CI: 0.72, 0.89) and robust external validation (AUC: 0.90, 95% CI: 0.86, 0.93; Cohen’s κ: 0.55, 95% CI: 0.45, 0.64). In our inter-rater analysis, the model achieved the highest average agreement (Cohen’s κ: 0.66, 95% CI: 0.57, 0.74), outperforming all nine pathologists whose Cohen’s κ ranged from 0.35 to 0.62. Limitations include the retrospective design and that the cross-scanner reproducibility and inter-rater analyses were conducted exclusively on internal validation data, potentially overestimating performance in these analyses.

**Conclusions:**

Our AI model demonstrates pathologist-level performance for cribriform morphology detection in prostate cancer. This approach could enhance diagnostic reliability, standardise reporting, and improve treatment decisions for patients with prostate cancer.

## Take Home Message

Cribriform morphology in prostate cancer indicates poor prognosis but remains underreported. Our artificial intelligence model achieved pathologist-level performance for cribriform detection in prostate biopsies. This could improve diagnostic consistency and standardise reporting of this prognostically important feature.

ADVANCING PRACTICE **What does this study add?**

This study presents the first comprehensively validated artificial intelligence system for detecting cribriform morphology in prostate cancer biopsies, achieving pathologist-level performance across multiple international cohorts and scanner platforms. When compared with nine expert uropathologists, the model showed the highest agreement among all raters. The system addresses a clinically critical yet frequently underreported feature that directly influences risk stratification and active surveillance eligibility and could improve diagnostic consistency in prostate cancer management.

**Clinical Relevance**

Cribriform growth is increasingly recognized as a key predictive factor in prostate cancer management, particularly in determining eligibility for active surveillance. Similar to advances in imaging, artificial intelligence has the potential to optimize pathology workflows, reduce interobserver variability, and support more informed treatment decisions. The current challenge lies in effectively implementing these innovations across routine pathology practice throughout Europe. Associate Editor: Roderick C.N. van den Bergh.

**Patient Summary**

In this study, we developed an artificial intelligence model to identify a specific high-risk subtype of prostate cancer known as cribriform. We found that our model performed as well as experienced pathologists. This technology could help doctors more reliably identify aggressive forms of prostate cancer and make better treatment decisions for patients.

## 1 Introduction

Cribriform morphology in prostate cancer indicates increased metastatic potential, and is associated with adverse outcomes and increased mortality [1], [2]. The term cribriform comes from the Latin *cribrum*, meaning *sieve*, which describes its appearance where malignant epithelial cells form sheets punctured by sieve-like spaces [3], [4]. By definition, cribriform morphology is classified as at least Gleason pattern 4 [3]. In core needle biopsies, the prevalence of cribriform morphology ranges from 4% (for Gleason 3 + 4) up to 21% (for higher grade tumours) [5], [6], [7]. Given its prognostic value, the presence of cribriform morphology now contraindicates active surveillance strategies in prostate cancer management [8], [9].

Despite this clinical importance, cribriform morphology remains underreported in routine practice [5]. This creates gaps in patient risk stratification. Furthermore, like Gleason grading, identifying cribriform patterns shows substantial interobserver variability and requires specialist expertise for consistent identification [7]. These diagnostic challenges are compounded by increasing workload pressures in pathology departments. Rising case volumes and declining number of specialists are stretching resources [10].

While artificial intelligence (AI) solutions have emerged to address workload challenges, current approaches fail to fully meet the spectrum of diagnostic needs. Many AI models for prostate cancer focus solely on Gleason score [11]. However, comprehensive pathological reporting requires additional features beyond Gleason scoring. An effective AI solution must recognise and report multiple pathological features from a biopsy, with cribriform morphology detection being particularly important.

Currently, no study has sufficiently validated an AI-based system for cribriform detection. This study therefore aims to develop and validate an AI model for the automatic detection of cribriform morphology in prostate core needle biopsies.

## 2 Materials and methods

**Fig. 1:**
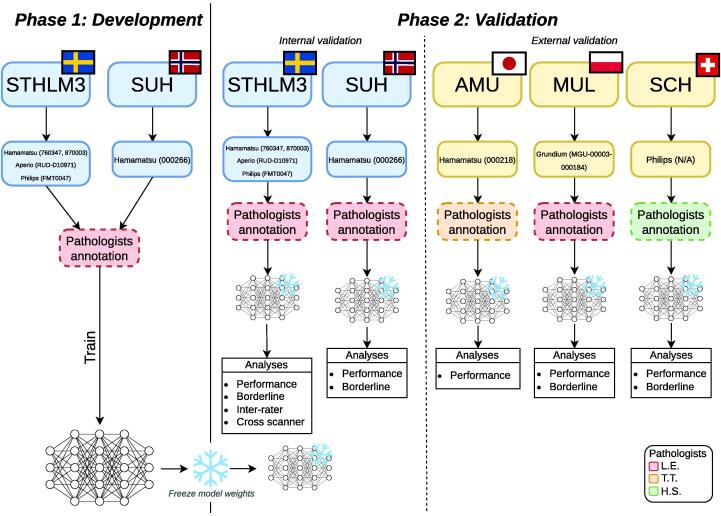
**Overview of the study design**. Phase 1 (Development) used subsets of the STHLM3 and SUH cohorts for model training. Phase 2 (Validation) included internal validation on reserved STHLM3/SUH data and external validation on three independent cohorts (AMU, MUL, SCH). Slides were digitised on scanners from multiple vendors and annotated by three pathologists. Numbers in parentheses indicate scanner serial numbers. Serial numbers for the scanners used at SCH are unavailable, but these scanners are distinct from those used in the other cohorts. No scanners in the external cohorts were present in the training data. Performance evaluation included standard metrics (AUC, Cohen’s κ, sensitivity, specificity), inter-rater analysis comparing our model with nine pathologists, cross-scanner reproducibility assessment, and borderline case analysis. Definition of abbreviations: AUC = area under the receiver operating characteristic curve.

We conducted a retrospective study in two phases: (1) model development and (2) validation (Fig. 1). The study protocol has been published [12].

### 2.1 Data and participants

We digitised formalin-fixed paraffin-embedded, haematoxylin and eosin-stained prostate core needle biopsy slides from six cohorts: the Stockholm3 (STHLM3) trial [13], Capio S:t Göran Hospital, Sweden (STG), Stavanger University Hospital, Norway (SUH), Aichi Medical University, Japan (AMU), Medical University of Lodz, Poland (MUL), and Synlab Switzerland (SCH). Complete information about the cohorts—including collection dates, participants, and sampling methods—can be found in the protocol [12]. The slides were digitised using eight scanner instruments from four different vendors, including Philips (STHLM3, SCH), Grundium (MUL), Hamamatsu (AMU, STHLM3, SUH), and Aperio (STHLM3, STG). Some slides were scanned multiple times using different scanners. As cribriform morphology occurs only in Gleason pattern ≥4 disease, all sampling for both training and evaluation was restricted to slides containing Gleason pattern 4 or 5. Please refer to Table 2 in the protocol for details regarding slide digitisation across cohorts [12].

Parts of the STHLM3, STG, and SUH cohorts were used for training and tuning the model during phase 1 (model development), while a portion of data from these cohorts was reserved for internal validation during phase 2 (model validation). The AMU, MUL, and SCH cohorts were used entirely for external validation during phase 2. The validation datasets used during phase 2 were completely independent from the development process in phase 1 and only used for validation once. In other words, after phase 1, the model remained entirely fixed (frozen) throughout phase 2 without any adjustments. We defined validation cohorts as “internal” when their laboratory and/or specific scanner instrument had been included in the development set, while “external” cohorts contained samples from physical scanner instruments and laboratories that were completely independent from those used in development. All data partitions used to separate development from validation sets were grouped at the patient level to prevent data leakage, ensuring that slides from the same patient never appeared in both training and validation datasets.

### 2.2 Outcome

Cribriform morphology was defined per International Society of Urological Pathology (ISUP) 2021 consensus as confluent malignant epithelial cells with multiple glandular lumina visible at low power (x10 objective), without intervening stroma or mucin between glandular structures [4]. Cribriform growth was annotated irrespective of whether it was invasive (within acinar adenocarcinoma) or noninvasive (intraductal carcinoma). This was justified by both forms often being assessed and reported together for prognostication and treatment planning, a practice supported by the 2019 ISUP consensus [14]. A slide was labelled as positive if any core contained any cribriform morphology as defined previously, irrespective of extent.

To minimise interobserver variability, we established a reference standard based on annotations from the lead pathologist (L.E.) or other experienced uropathologists (H.S., T.T.) whose concordance has been quantified in earlier studies [7]. To reduce the annotation burden for the reference standard pathologists, nonreference standard pathologists initially reviewed cases with Gleason pattern 4 to identify suspect slides with cribriform morphology. These preliminary annotations were used to upsample suspect cribriform cases for subsequent reference standard annotation. A nonreference standard pathologist was defined as one whose concordance to the lead pathologist (L.E.) is unknown. Nonreference standard annotations were not used to assess model performance.

For the STHLM3 and STG cohorts, a collection of 700 slides containing Gleason pattern 4 was assessed and annotated by the lead pathologist. In the other cohorts (SUH, MUL, and SCH), initial annotations were made by nonreference standard pathologists. A sample, with positive cases upsampled, was re-labelled by a reference standard pathologist. Detailed annotation protocols for each cohort are provided in Table A1 and the protocol [12].

For STHLM3 and STG, pixel-level annotations for glands representing cribriform morphology were made. For the other cohorts, only slide-level labels were annotated. When establishing the reference standard, L.E. (on STHLM3, SUH, and MUL) and H.S. (on SCH) also indicated cases they considered borderline cribriform. The term borderline was used for cases with features suggestive of cribriform growth that did not fully meet established morphological criteria. This category was intended to capture diagnostically difficult cases to permit statistical analyses on this specific substratum.

For a subset of the STHLM3 internal validation data, we also have annotations from the nine expert uropathologists included in an earlier interobserver reproducibility study [7].

### 2.3 Model development

We extracted smaller images, referred to as patches, from each whole-slide image (WSI) for input into the model. Each patch measured 256 × 256 pixels at 1 μm per pixel (10× magnification) and overlapped with neighbouring patches by 50% both vertically and horizontally. Patches with tissue covering <10% of the image were discarded based on tissue segmentation masks. An in-house segmentation model built on UNet++ with a ResNeXt-101 (32 × 4d) encoder was used to create the tissue segmentation masks [15].

We developed a multiple instance learning model using an EfficientNetV2-S neural network backbone to detect cribriform morphology. The model processes WSIs by using the extracted patches, treating each slide as a bag of patches. These patches are processed through the neural network to extract patch-level features, which are aggregated via a gated attention mechanism to create slide-level features. To enhance generalisability, we implemented extensive data augmentation techniques. The final model used an ensemble of 10 models trained during 10-fold cross-validation, and used test-time augmentation for final predictions. Further details are available in the supplement (Section B).

### 2.4 Statistical analysis

All analyses were prespecified [12]. We performed analyses at both individual cohort levels and aggregated internal and external cohort levels. Model performance was evaluated using receiver operating characteristic (ROC) curves and the area under the ROC curve (AUC). An operating point of 0.5 was used for binary classification. We calculated sensitivity and specificity. We also measured the agreement between the model and the reference standard using Cohen’s κ. For glass slides that were digitised multiple times in the STHLM3 cohort, performance metrics were calculated using only the original WSI that was annotated by the pathologist, rather than including all digital copies of the same physical slide. The 95% confidence intervals (CIs) for all metrics were calculated from nonparametric bootstrapping using 1000 bootstrap samples. We created visual calibration plots to assess model calibration.

Using annotations on a subset of STHLM3 data from nine pathologists and our model, we conducted an inter-rater variability analysis to compare the model’s performance against expert pathologists. For each rater, including our model, we calculated the mean pairwise Cohen’s κ coefficient against the other pathologists to quantify agreement levels. Furthermore, we conducted sensitivity analyses to evaluate cross-scanner reproducibility by calculating the pairwise Cohen’s κ between model predictions on different digital scans of the same glass slides. This analysis used slides from the STHLM3 cohort that had been digitised multiple times using scanners from four vendors (Aperio, Grundium, Hamamatsu, Philips). Lastly, in an exploratory analysis, we quantified the prevalence of “borderline” cases in both true negative and false-positive groups using annotations from L.E. and H.S. In analyses not specifically focused on borderline cases, these were classified as negative.

## 3 Results

### 3.1 Dataset characteristics

Patient and slide characteristics are summarised in Table 1 and A5. The study included a total of 705 patients: 430 in the training set, 171 in the internal validation set, and 104 in the external validation set (Table A4). Training was done on 1280 WSIs from 640 physical slides. The internal validation cohorts included 211 physical slides from STHLM3 and 50 from SUH. The external validation cohorts contained 137 slides from MUL and 56 from SCH. The prevalence of cribriform pattern was higher in the external validation set (35%, *n* = 94) compared to training (24%, *n* = 155) and internal validation sets (24%, *n* = 62). The most common age interval was 65–69 yr, comprising 40% of patients. Gleason score and ISUP grade distributions were relatively consistent across training, internal validation, and external validation sets.

**Table 1:** Patient and slide characteristics across all cohorts, showing demographic and clinical data

**Cohort**	**STG**	**STHLM3**		**SUH**		**AMU**	**MUL**	**SCH**
**Split**	**Train**	**Train**	**Test**	**Train**	**Test**	**Test**	**Test**	**Test**
**Patients**								
***n***	67	287	140	76	31	43	49	12
**Age, yr**								
*≤*49	0 (0%)	0 (0%)	1 (*<*1%)	0 (0%)	0 (0%)	0 (0%)	1 (2%)	0 (0%)
50–54	1 (2%)	17 (6%)	6 (4%)	3 (4%)	1 (3%)	0 (0%)	1 (2%)	0 (0%)
55–59	2 (4%)	31 (11%)	18 (13%)	3 (4%)	2 (6%)	0 (0%)	1 (2%)	3 (25%)
60–64	4 (9%)	80 (28%)	35 (25%)	14 (18%)	1 (3%)	0 (0%)	6 (12%)	3 (25%)
65–69	4 (9%)	149 (52%)	72 (51%)	14 (18%)	6 (19%)	0 (0%)	9 (18%)	3 (25%)
*≥*70	36 (77%)	10 (3%)	8 (6%)	42 (55%)	21 (68%)	0 (0%)	31 (63%)	3 (25%)
Missing	20	0	0	0	0	43	0	0
**PSA, ng/mL**								
*<*3	3 (7%)	45 (16%)	16 (11%)	4 (5%)	0 (0%)	1 (2%)	0 (0%)	0 (0%)
3–*<*5	0 (0%)	88 (31%)	56 (40%)	5 (7%)	1 (3%)	1 (2%)	0 (0%)	1 (11%)
5–*<*10	7 (16%)	76 (26%)	39 (28%)	34 (45%)	13 (42%)	11 (26%)	0 (0%)	4 (44%)
*≥*10	35 (78%)	78 (27%)	29 (21%)	32 (43%)	17 (55%)	30 (70%)	0 (0%)	4 (44%)
Missing	22	0	0	1	0	0	49	3
**Whole-slide images**								
***n***^a^	79	1,051	608	150	50	73	137	56
**Physical slides**	79	411	211	150	50	73	137	56
**Cribriform**	27 (34%)	81 (20%)	43 (20%)	47 (31%)	19 (38%)	28 (38%)	55 (40%)	11 (20%)
Gleason score								
3 + 3	0 (0%)	0 (0%)	0 (0%)	0 (0%)	0 (0%)	0 (0%)	0 (0%)	3 (5%)
3 + 4	0 (0%)	61 (15%)	25 (12%)	68 (45%)	13 (26%)	0 (0%)	21 (15%)	18 (32%)
3 + 5	1 (1%)	11 (3%)	1 (*<*1%)	0 (0%)	0 (0%)	0 (0%)	0 (0%)	5 (9%)
4 + 3	2 (3%)	131 (32%)	74 (35%)	37 (25%)	16 (32%)	0 (0%)	38 (28%)	19 (34%)
4 + 4	17 (22%)	158 (38%)	73 (35%)	25 (17%)	15 (30%)	0 (0%)	35 (26%)	5 (9%)
4 + 5	27 (34%)	39 (9%)	34 (16%)	18 (12%)	4 (8%)	0 (0%)	28 (20%)	6 (11%)
5 + 3	0 (0%)	1 (*<*1%)	0 (0%)	0 (0%)	0 (0%)	0 (0%)	0 (0%)	0 (0%)
5 + 4	19 (24%)	6 (1%)	2 (*<*1%)	2 (1%)	2 (4%)	0 (0%)	15 (11%)	0 (0%)
5 + 5	13 (16%)	4 (*<*1%)	2 (*<*1%)	0 (0%)	0 (0%)	0 (0%)	0 (0%)	0 (0%)
Missing	0	0	0	0	0	73	0	0
**ISUP**								
1	0 (0%)	0 (0%)	0 (0%)	0 (0%)	0 (0%)	0 (0%)	0 (0%)	3 (5%)
2	0 (0%)	61 (15%)	25 (12%)	68 (45%)	13 (26%)	0 (0%)	21 (15%)	18 (32%)
3	2 (3%)	131 (32%)	74 (35%)	37 (25%)	16 (32%)	0 (0%)	38 (28%)	19 (34%)
4	18 (23%)	170 (41%)	74 (35%)	25 (17%)	15 (30%)	0 (0%)	35 (26%)	10 (18%)
5	59 (75%)	49 (12%)	38 (18%)	20 (13%)	6 (12%)	0 (0%)	43 (31%)	6 (11%)
Missing	0	0	0	0	0	73	0	0

PSA = prostate-specific antigen; ISUP = International Society of Urological Pathology Grade.

a Total number of whole slide images (digital copies of physical slides). This may exceed the number of physical slides when slides from a cohort were scanned multiple times on different scanners.

**Table 2:** Performance metrics across all cohorts, including AUC, Cohen’s kappa, sensitivity, and specificity values with 95% confidence intervals. Type indicates the cohort’s validation status

**Cohort**	**Type**	**AUC**	**Cohen’s κ**	**Sensitivity**	**Specificity**
STHLM3	Internal	0.96 (0.94, 0.99)	0.8 (0.69, 0.9)	0.88 (0.78, 0.98)	0.95 (0.91, 0.98)
SUH	Internal	0.98 (0.95, 1.0)	0.8 (0.63, 0.96)	1.0 (1.0, 1.0)	0.84 (0.7, 0.97)
AMU	External	0.92 (0.86, 0.97)	0.42 (0.27, 0.6)	1.0 (1.0, 1.0)	0.49 (0.33, 0.63)
MUL	External	0.89 (0.83, 0.94)	0.53 (0.39, 0.65)	0.89 (0.8, 0.97)	0.67 (0.56, 0.77)
SCH	External	0.95 (0.87, 0.99)	0.71 (0.4, 0.93)	0.73 (0.43, 1.0)	0.96 (0.89, 1.0)
Overall	Internal	0.97 (0.95, 0.99)	0.81 (0.72, 0.89)	0.92 (0.85, 0.98)	0.93 (0.89, 0.96)
Overall	External	0.9 (0.86, 0.93)	0.55 (0.45, 0.64)	0.9 (0.84, 0.96)	0.7 (0.63, 0.76)

AUC = area under the receiver operating characteristic curve. AMU = Aichi Medical University, Japan. MUL = Medical University of Lodz, Poland. SCH = Synlab Switzerland. STG = Capio S:t Göran Hospital, Sweden. STHLM3 = Stockholm3 trial. SUH = Stavanger University Hospital, Norway.

### 3.2 Model performance

**Fig. 2:**
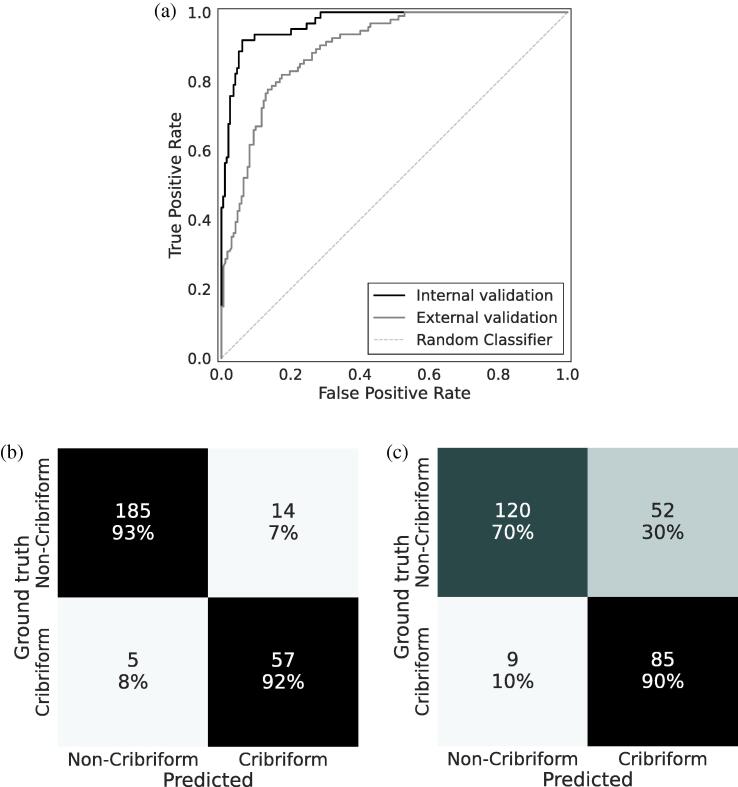
Model performance in discriminating cribriform from non-cribriform prostate cancer across internal and external validation cohorts**. (A)** Receiver operating characteristic curves showing model performance on internal and external validation sets. **(B)** Confusion matrix on predictions for the internal validation set (STHLM3 and SUH). **(C)** Confusion matrix on predictions for the external validation set (AMU, MUL, and SCH).

On the internal validation set (STHLM3 and SUH cohorts), our deep learning model demonstrated an AUC of 0.97 (95% CI: 0.95, 0.99) and a Cohen’s κ of 0.81 (95% CI: 0.72, 0.89), with a sensitivity of 0.92 (95% CI: 0.85, 0.98) and specificity of 0.93 (95% CI: 0.89, 0.96). For external validation (AMU, MUL, and SCH), the model achieved an AUC of 0.90 (95% CI: 0.86, 0.93) and a Cohen’s κ of 0.55 (95% CI: 0.45, 0.64), with a sensitivity of 0.90 (95% CI: 0.84, 0.96) and specificity of 0.70 (95% CI: 0.63, 0.76). The ROC curves and confusion matrices illustrating these performance differences are presented in Fig. 2 and A2, while AUC, Cohen’s κ, sensitivity, and specificity for all included cohorts are presented in Table 2.

Examining individual cohorts (Table 2), performance varied across datasets. STHLM3 achieved an AUC of 0.96 (95% CI: 0.94, 0.99) and a Cohen’s κ of 0.80 (95% CI: 0.69, 0.90), while SUH demonstrated similar results with an AUC of 0.98 (95% CI: 0.95, 1.0) and a similar Cohen’s κ of 0.80 (95% CI: 0.63, 0.96). Performance in external validation cohorts was more variable. The SCH cohort maintained results comparable to internal validation, with an AUC of 0.95 (95% CI: 0.87, 0.99) and a Cohen’s κ of 0.71 (95% CI: 0.40, 0.93). However, while the AMU and MUL cohorts preserved good discriminative ability with AUCs of 0.92 (95% CI: 0.86, 0.97) and 0.89 (95% CI: 0.83, 0.94) respectively, their agreement metrics were notably lower, with Cohen’s κ values of 0.42 (95% CI: 0.27, 0.60) for AMU and 0.53 (95% CI: 0.39, 0.65) for MUL.

The model showed good calibration internally, with predicted probabilities closely matching observed cribriform morphology (Fig. A3). In external validation, calibration deviated—especially at intermediate probabilities—leading to overdiagnosis and reduced specificity at the same operating point (0.5) used on the internal validation sets (Fig. 2 and A1).

In our exploratory analysis of borderline cases (Table A8), using annotations from L.E. (on STHLM3, SUH, and MUL) and H.S. (SCH), we found a significantly higher proportion of borderline cases among false positive cases (38%) compared to true negative cases (14%; Fisher’s exact test, p=0.008). In the cross-scanner reproducibility analysis all scanners showed high concordance, with pairwise agreement ranging from 0.90 to 0.97. Scanner specific results for the cross-scanner reproducibility analysis are presented in the supplementary material (Section C).

### 3.3 Comparison with pathologists

**Fig. 3:**
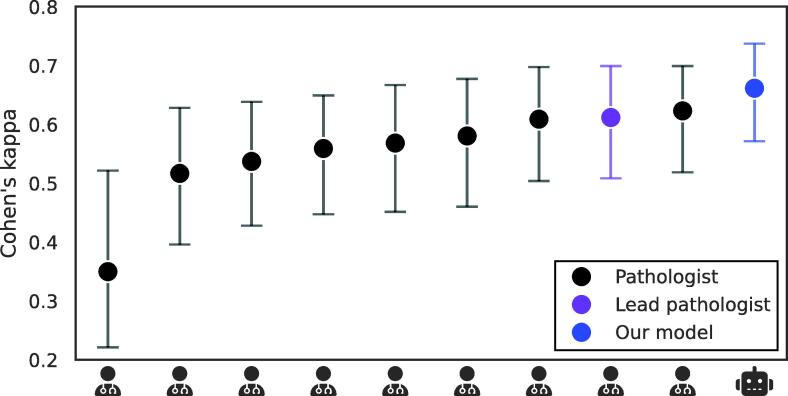
**Pathologist concordance analysis.** Agreement between our model (robot icon) and nine pathologists (physician icon), showing mean pairwise Cohen’s κ values. For each rater, including our model, the mean pairwise Cohen’s κ was calculated against the other pathologists only (the model was excluded from this average calculation). The whiskers indicate the 95% confidence interval. For exact values see Table A6.

In the inter-rater analysis (Fig. 3 and Table A6) we compared the model with nine pathologists on a subset of 88 slides from the STHLM3 validation cohort. Using the lead pathologist’s (L.E.) annotations as a reference, 43 slides were positive for the cribriform pattern. The model achieved the highest average pairwise Cohen’s κ of 0.66 (95% CI: 0.57, 0.74). This exceeded the performance of all nine pathologists, whose average pairwise Cohen’s κ values ranged from 0.35 (95% CI: 0.22, 0.52) to 0.62 (95% CI: 0.52, 0.70). The lead pathologist, who annotated the training data, ranked third with an average pairwise Cohen’s κ of 0.61 (95% CI: 0.51, 0.7).

## 4 Discussion

In this study, we developed and validated a deep learning model to detect cribriform morphology in prostate cancer biopsies. Our model demonstrated strong discriminative performance across both internal and external cohorts, achieving high agreement with experienced pathologists’ annotations. The intended workflow of our model is to apply it only after Gleason pattern 4 or 5 has been identified (eg, by routine pathology review or an upstream AI model for cancer detection and grading), where it can serve as a second-reader/triage tool to flag cases and regions suspicious for cribriform morphology.

In the inter-rater analysis, the model achieved the highest average agreement when compared against nine expert pathologists. Importantly, the model was trained on annotations from a single pathologist rather than a consensus panel; however, generalised to achieve the highest concordance with the broader group, surpassing even the training annotator, who ranked third. This result should not be directly interpreted as diagnostic superiority over individual pathologists, but rather as evidence that the model has learned a consistent representation of cribriform morphology that aligns well with expert opinion.

Although the model maintained strong discriminative ability externally (AUC: 0.90), specificity decreased from 93% internally to 70% externally, and Cohen’s κ decreased from 0.81 to 0.55. Several factors contribute to this performance gap. First, the enrichment sampling strategy used to assemble validation cohorts upsamples cases that a nonreference-standard pathologist considered positive for cribriform morphology. When the reference standard pathologist subsequently reclassifies a proportion of these as negative, the resulting negative class is enriched for morphologically ambiguous cases, as there was sufficient cribriform-like morphology to prompt the initial positive assessment. This concentrates diagnostically challenging cases among the negatives and alters class proportions relative to an unselected Gleason pattern 4+ population. Furthermore, the different upsampling strategies lead to differing class proportions. This affects prevalence-dependent metrics, such as Cohen’s κ, and contributes to the calibration drift seen. Second, applying a fixed decision threshold (0.5) across cohorts that differ in a multitude of characteristics means that the same threshold yields different sensitivity–specificity trade-offs in different settings. Third, interobserver variability in cribriform assessment itself likely contributes, as prior work shows only moderate agreement among expert uropathologists (mean Cohen’s κ 0.56) [7]. The three external test sets were annotated by three different pathologists.

The borderline case analysis further contextualises these findings, roughly 40% of the model’s false positives were considered borderline cribriform by the annotating pathologists. In clinical practice, borderline cases are not reported as cribriform and are effectively treated as negative, a convention our primary analyses mirror. This conservative classification means that model predictions flagging borderline cases are counted as false positives, which may inflate the apparent false-positive rate. However, in the model’s intended role as a triage tool, flagging diagnostically ambiguous cases for expert review is a desirable property, and the true classification error rate may therefore be lower than the reported metrics suggest. Importantly, despite these shifts, the model’s sensitivity remained consistently high across cohorts (0.90–0.92), which is the more clinically relevant metric for a triage application where the cost of over-flagging is modest relative to the cost of missing cribriform morphology that could lead to inappropriate active surveillance.

A strength of our study is the comprehensive validation strategy, which employed both internal and external validation cohorts alongside inter-rater and cross-scanner reproducibility analyses. However, some limitations must be acknowledged. Both the cross-scanner reproducibility and pathologist concordance analyses were conducted on internal validation sets, potentially overestimating performance. These analyses were enabled by the availability of multi-rater annotations and repeatedly scanned slides from prior studies exclusively on the STHLM3 cohort. Additionally, the model was evaluated only on slides containing high-grade cancer (Gleason pattern 4 or 5), which, while not an apparent limitation in relation to its intended use, is important to note.

To our knowledge, this is the first study to comprehensively validate an AI model specifically for cribriform pattern detection in prostate cancer. Previous research has primarily focused on Gleason grading or tumour detection [11]. Two earlier studies featured models for cribriform detection, but showed only modest results and lacked external multi-cohort validation [16], [17]. Our approach advances this work through validation across multiple external, international cohorts and comparison of model performance against multiple pathologists.

The accurate detection of cribriform morphology represents one of the crucial decision points in treatment planning for patients with prostate cancer. This prognostically significant pattern, though often overlooked in practice, directly influences risk stratification and treatment selection. Our model attempts to address this challenge by providing support for pathologists and enhancing diagnostic consistency. For pathologists confronting mounting caseloads, the model could offer assistance by highlighting regions of interest, streamlining workflows, and supporting diagnostic decisions. Improved reliability in cribriform detection could translate to better-informed treatment assessments for patients.

We see the next step being the integration of the cribriform detection model with models for Gleason grading, cancer extent estimation, and detection of perineural invasion as components of an overall system enabling prospective evaluation. To combat the calibration drift seen in the current study, we would expect a site-specific recalibration strategy to be needed in deployment, with conformal prediction offering a potential solution [18]. Clinical deployment will require regulatory clearance (eg, CE-IVDR) and integration into routine pathology systems, which remains a limiting factor as digital pathology is not yet universally implemented across Europe [19].

## 5 Conclusion

Our deep learning model demonstrates robust performance for automated cribriform morphology detection in prostate cancer, with performance comparable to experienced pathologists. This approach could enhance diagnostic reliability, standardise reporting of this prognostically important feature, and potentially improve treatment decisions for patients with prostate cancer.

  ***Author contributions:*** Kelvin Szolnoky had full access to all the data in the study and takes responsibility for the integrity of the data and the accuracy of the data analysis.

  *Study concept and design:* Egevad, Eklund, Kartasalo.

*Acquisition of data:* Blilie, Tsuzuki, Samaratunga, Titus, Gudlaugsson, Kjosavik, Asenjo, Gambacorta, Libretti, Braun, Kordek, Lowicki, Delahunt, Iczkowski, van der Kwast, van Leenders, Leite, Pan, Janssen, Egevad.

*Analysis and interpretation of data:* Szolnoky, Kartasalo.

*Drafting of the manuscript:* Szolnoky.

*Critical revision of the manuscript for important intellectual content:* All authors.

*Statistical analysis:* Szolnoky.

*Obtaining funding*: Blilie, Eklund, Kartasalo, Egevad.

*Administrative, technical, or material support*: Szolnoky, Mulliqi, Ji, Boman.

*Supervision:* Kartasalo, Eklund.

*Other* (specify): None.

  ***Financial disclosures:*** Kelvin Szolnoky certifies that all conflicts of interest, including specific financial interests and relationships and affiliations relevant to the subject matter or materials discussed in the manuscript (eg, employment/affiliation, grants or funding, consultancies, honoraria, stock ownership or options, expert testimony, royalties, or patents filed, received, or pending), are the following: Nita Mulliqi, Martin Eklund, Lars Egevad and Kimmo Kartasalo are shareholders of Clinsight AB. Anders Blilie received a grant from the Health Faculty at the University of Stavanger, Norway. Martin Eklund received funding from the Swedish Research Council, Swedish Cancer Society, Swedish Prostate Cancer Society, Nordic Cancer Union, Karolinska Institutet, and Region Stockholm. Kimmo Kartasalo received funding from the SciLifeLab & Wallenberg Data Driven Life Science Program (KAW 2024.0159), David and Astrid Hägelen Foundation, Instrumentarium Science Foundation, KAUTE Foundation, Karolinska Institute Research Foundation, Orion Research Foundation and Oskar Huttunen Foundation. Lars Egevad received funding from the Swedish Cancer Foundation (23 2641 Pj) and the Stockholm Cancer Society (234053). The other authors declare no potential conflicts of interest.

  ***Funding/Support and role of the sponsor*:** None.

  ***Acknowledgements:*** The computations were made possible through the National Academic Infrastructure for Supercomputing in Sweden (NAISS) and the Swedish National Infrastructure for Computing (SNIC) at C3SE partially funded by the Swedish Research Council through grant agreement no. 2022-06725 and no. 2018-05973, and by the supercomputing resource Berzelius provided by the National Supercomputer Centre at Linköping University and the Knut and Alice Wallenberg Foundation.

  ***Data and code availability statement:*** The clinical data and whole slide images used in this study are not publicly available due to patient privacy considerations and ethical restrictions. Raw outcome predictions from the AI model are available from the corresponding author upon reasonable request. The code developed for this study is not available.

  ***Ethical considerations:*** The study is conducted in agreement with the Declaration of Helsinki. The data were retrieved in ≥1 rounds at each of the participating international sites between May 1, 2012, and May 1, 2024. All data were deidentified at each site and provided to Karolinska Institutet in anonymised format. The centralised collection of patient samples from the international sites to Karolinska Institutet was approved by the Swedish Ethical Review Authority (permit 2019-05220). The following local approvals were provided to cover the data collection at each site: AMU (permit 2023-074 for the AMU cohort), Stockholm regional ethics committee (permits 2012/572-31/1, 2012/438-31/3, and 2018/845-32 for the STG and STHLM3 cohorts), the Bioethics Committee at the Medical University of Lodz (permit RNN/295/19/KE for the MUL cohort), and the Regional Committee for Medical and Health Research Ethics (REC) in Western Norway (permits REC/Vest 80924, REK 2017/71 for the SUH cohort). For the SCH cohort, ethical approval was waived by the respective local institutional review boards due to the retrospective usage of fully deidentified prostate specimens, and the data collection under the waiver was approved by the Swedish Ethical Review Authority (permit 2019-05220). Written informed consent was provided by the participants in the STHLM3 dataset.

  We want to thank Carin Cavalli-Björkman for assistance with scanning and database support. We would also like to thank Silja Kavlie Fykse and Desmond Mfua Abono for scanning in Stavanger. We would like to acknowledge the patients who participated in the STHLM3 diagnostic study and the OncoWatch and NordCaP projects and contributed the clinical information that made this study possible.
