# Supplementary material for: Finding Holes: Pathologist-Level Performance Using AI for Cribriform Morphology Detection in Prostate Cancer
Source: Eur Urol Open Sci. 2026 Apr 7;87:31–9. doi: 10.1016/j.euros.2026.03.016 (PMC13090957; doi:10.1016/j.euros.2026.03.016)
Supplement: Supplementary Data 1 [file mmc1.pdf]

SUPPLEMENTARY MATERIAL

FINDING HOLES: PATHOLOGIST LEVEL PERFORMANCE USING AI FOR CRIBRIFORM MORPHOLOGY DETECTION IN PROSTATE CANCER

Kelvin Szolnoky[a], Anders Blilie[b,c], Nita Mulliqi[a], Toyonori Tsuzuki[d], Hemamali Samaratunga[e], Matteo Titus[a], Xiaoyi Ji[a], Sol Erika Boman[a,f], Einar Gudlaugsson[b], Svein Reidar Kjosavik[g,h], José Asenjo[i], Marcello Gambacorta[j], Paolo Libretti[j], Marcin Braun[k], Radzisław Kordek[l], Roman Łowicki[l], Brett Delahunt[m,n], Kenneth A. Iczkowski[o], Theo van der Kwast[p], Geert J. L. H. van Leenders[q], Katia R. M. Leite[r], Chin-Chen Pan[s], Emiel Adrianus Maria Janssen[b,t,u], Martin Eklund[a], Lars Egevad[n], and Kimmo Kartasalo[v]

[a]Department of Medical Epidemiology and Biostatistics, Karolinska Institutet, Stockholm, Sweden
[b]Department of Pathology, Stavanger University Hospital, Stavanger, Norway
[c]Faculty of Health Sciences, University of Stavanger, Stavanger, Norway
[d]Department of Surgical Pathology, School of Medicine, Aichi Medical University, Nagoya, Japan
[e]Aquesta Uropathology and University of Queensland, Brisbane, Queensland, Australia
[f]Department of Molecular Medicine and Surgery, Karolinska Institutet, Stockholm, Sweden
[g]The General Practice and Care Coordination Research Group, Stavanger University Hospital, Stavanger, Norway
[h]Department of Global Public Health and Primary Care, Faculty of Medicine, University of Bergen, Bergen, Norway
[i]Department of Pathology, SYNLAB, Madrid, Spain
[j]Department of Pathology, SYNLAB, Brescia, Italy
[k]Department of Pathology, Chair of Oncology, Medical University of Lodz, Lodz, Poland
[l]1st Department of Urology, Medical University of Lodz, Lodz, Poland
[m]Malaghan Institute of Medical Research, Wellington, New Zealand
[n]Department of Oncology and Pathology, Karolinska Institutet, Stockholm, Sweden
[o]Department of Pathology and Laboratory Medicine, University of California - Davis Health, Sacramento, CA, USA
[p]Laboratory Medicine Program and Princess Margaret Cancer Center, University Health Network, University of Toronto, Toronto, ON, Canada
[q]Department of Pathology, Erasmus MC, University Medical Center, Rotterdam, the Netherlands
[r]Department of Urology, Laboratory of Medical Research, University of São Paulo Medical School, Sao Paulo, Brazil
[s]Department of Pathology and Laboratory Medicine, Taipei Veterans General Hospital, Taipei, Taiwan
[t]Department of Chemistry, Bioscience and Environmental Engineering, University of Stavanger, Stavanger, Norway
[u]Institute for Biomedicine and Glycomics, Griffith University, Brisbane, Queensland, Australia
[v]Department of Medical Epidemiology and Biostatistics, SciLifeLab, Karolinska Institutet, Stockholm, Sweden

A Figures and Tables

Table A1. Labelling and sampling methodology across different cohorts, sampling strategies, and the annotating pathologist (reference standard).

Cohort	Initial sampling*	Initial annotator	Second sampling*	Reference standard
STHLM3/STG	701 slides containing GP 4	L.E.	N/A	L.E.
SUH	332 slides containing GP 4	A.B.	$120^{+}/40^{\text{borderline}}/40^{-}$ slides	L.E.
AMU	73 slides containing GP 4	T.T.	N/A	T.T.
MUL	276 slides containing GP 4	A.B.	$74^{+}/63^{-}$ slides	L.E.
SCH	56 slides containing GP 4	Site pathologists	$12^{+}/6^{-}$ blocks	H.S.

Definition of abbreviations: GP 4 = Gleason pattern 4.

* To enhance statistical power and reduce the annotation burden for the reference standards, a two-stage enrichment sampling strategy was employed. Initially, a non-reference standard pathologist annotated slides containing Gleason pattern 4. Subsequently, slides were resampled based on these preliminary annotations to enrich for potential cribriform patterns before final annotation by the reference standard pathologist. In the *second sampling* column, superscript symbols indicate the initial pathologist's assessment for cribriform and how sampling was done based off of these annotations. In the STHLM3, STG, and AMU cohorts the initial annotator was the reference standard, i.e. no second round of annotations was needed.

Table A2. Hyperparameters for the patch level model.

Hyperparameter	Value
Encoder	EfficientNetV2-S
Initial weights	Weights from Gleason scoring encoder
Loss function	Binary cross entropy loss (weighted)
Optimiser	AdamW
Learning rate	OneCycleLR scheduler (starting at $1 \cdot 10^{-5}$, peaking at $1 \cdot 10^{-4}$ after 1 epoch, and finally decreasing to $1 \cdot 10^{-6}$ following a cosine annealing schedule)
Weight decay	$1 \cdot 10^{-2}$
Batch size	64
Precision	bfloat16
Train augmentations	Random: crop, horizontal and vertical flip, 90 degrees rotation, colour jitter, gamma, tone curve, grey scale, unsharp mask or guassian blur, ISO noise, gaussian noise, multiplicative noise, JPEG compression

Table A3. Hyperparameters for the slide level model.

Hyperparameter	Value
Encoder	EfficientNetV2-S
Initial weights	Cribriform patch level weights
Loss function	Binary cross entropy loss (weighted)
Optimiser	RAdam
Learning rate	$3 \cdot 10^{-5}$ (constant)
Weight decay	$1 \cdot 10^{-5}$
Batch size	1
Max bag size	2200
Precision	bfloat16
Train augmentations	Random: crop, horizontal and vertical flip, 90 degrees rotation, colour jitter, gamma, tone curve, grey scale, unsharp mask or gaussian blur, ISO noise, gaussian noise, multiplicative noise, JPEG compression
Test augmentations	Random: horizontal and vertical flip, 90 degrees rotation

Table A4. Patient and slide characteristics stratified by dataset split (training, internal validation, and external validation).

**Split**	**Train**	**Internal test**	**External test**	**Overall**
**Patients**				
n	430	171	104	705
**Age, years**				
≤49	0 (0%)	1 (<1%)	1 (2%)	2 (<1%)
50-54	21 (5%)	7 (4%)	1 (2%)	29 (5%)
55-59	36 (9%)	20 (12%)	4 (7%)	60 (9%)
60-64	98 (24%)	36 (21%)	9 (15%)	143 (22%)
65-69	167 (41%)	78 (46%)	12 (20%)	257 (40%)
≥70	88 (21%)	29 (17%)	34 (56%)	151 (24%)
Missing	20	0	43	63
**PSA, ng/mL**				
<3	52 (13%)	16 (9%)	1 (2%)	69 (11%)
3-<5	93 (23%)	57 (33%)	2 (4%)	152 (24%)
5-<10	117 (29%)	52 (30%)	15 (29%)	184 (29%)
≥10	145 (36%)	46 (27%)	34 (65%)	225 (36%)
Missing	23	0	52	75
**Whole Slide Images**				
n***	1,280	658	266	2,204
**Physical slides**	640	261	266	1,167
**Cribriform**	155 (24%)	62 (24%)	94 (35%)	311 (27%)
**Gleason score**				
3 + 3	0 (0%)	0 (0%)	3 (2%)	3 (<1%)
3 + 4	129 (20%)	38 (15%)	39 (20%)	206 (19%)
3 + 5	12 (2%)	1 (<1%)	5 (3%)	18 (2%)
4 + 3	170 (27%)	90 (34%)	57 (30%)	317 (29%)
4 + 4	200 (31%)	88 (34%)	40 (21%)	328 (30%)
4 + 5	84 (13%)	38 (15%)	34 (18%)	156 (14%)
5 + 3	1 (<1%)	0 (0%)	0 (0%)	1 (<1%)
5 + 4	27 (4%)	4 (2%)	15 (8%)	46 (4%)
5 + 5	17 (3%)	2 (<1%)	0 (0%)	19 (2%)
Missing	0	0	73	73
**ISUP**				
1	0 (0%)	0 (0%)	3 (2%)	3 (<1%)
2	129 (20%)	38 (15%)	39 (20%)	206 (19%)
3	170 (27%)	90 (34%)	57 (30%)	317 (29%)
4	213 (33%)	89 (34%)	45 (23%)	347 (32%)
5	128 (20%)	44 (17%)	49 (25%)	221 (20%)
Missing	0	0	73	73

* Total number of whole slide images (digital copies of physical slides). This may exceed the number of physical slides when slides from a cohort were scanned multiple times on different scanners.

Table A5. Patient and slide characteristics stratified by cribriform morphology status.

	**Cribriform**	**Non-cribriform**
**Patients**		
n	221	587
**Age, years**		
≤49	1 (<1%)	1 (<1%)
50-54	5 (3%)	27 (5%)
55-59	11 (6%)	54 (10%)
60-64	43 (22%)	123 (23%)
65-69	72 (37%)	219 (40%)
≥70	64 (33%)	120 (22%)
Missing	25	43
**PSA, ng/mL**		
<3	14 (8%)	60 (11%)
3-<5	26 (14%)	135 (26%)
5-<10	42 (23%)	165 (31%)
≥10	103 (56%)	166 (32%)
Missing	36	61
**Whole Slide Images**		
n*[*]*	544	1,660
**Physical slides**	311	856
**Gleason score**		
3 + 3	0 (0%)	3 (<1%)
3 + 4	17 (6%)	189 (23%)
3 + 5	3 (1%)	15 (2%)
4 + 3	86 (30%)	231 (28%)
4 + 4	112 (40%)	216 (27%)
4 + 5	53 (19%)	103 (13%)
5 + 3	0 (0%)	1 (<1%)
5 + 4	12 (4%)	34 (4%)
5 + 5	0 (0%)	19 (2%)
Missing	28	45
**ISUP**		
1	0 (0%)	3 (<1%)
2	17 (6%)	189 (23%)
3	86 (30%)	231 (28%)
4	115 (41%)	232 (29%)
5	65 (23%)	156 (19%)
Missing	28	45

[*] Total number of whole slide images (digital copies of physical slides). This may exceed the number of physical slides when slides from a cohort were scanned multiple times on different scanners.

Table A6. Mean pairwise Cohen's kappa values for our model and nine pathologists, evaluating 88 slides (43 annotated cribriform-positive by the lead pathologist) from the STHLM3 cohort. For each rater, including our model, the average was calculated against the pathologists only (the model was excluded from this average calculation). Values in parentheses indicate the 95% confidence interval.

Rater	Cohen's kappa
**Our model**	**0.66 (0.57, 0.74)**
Pathologist 1	0.62 (0.52, 0.7)
**Lead pathologist**	**0.61 (0.51, 0.7)**
Pathologist 3	0.61 (0.5, 0.7)
Pathologist 4	0.58 (0.46, 0.68)
Pathologist 5	0.57 (0.45, 0.67)
Pathologist 6	0.56 (0.45, 0.65)
Pathologist 7	0.54 (0.43, 0.64)
Pathologist 8	0.52 (0.4, 0.63)
Pathologist 9	0.35 (0.22, 0.52)

Table A7. Cross-scanner reproducibility analysis showing pairwise Cohen's kappa values between different scanner types for 71 slides (19 annotated cribriform-positive by the lead pathologist) from the STHLM3 validation set that were scanned on 4 different scanners. Values in parentheses indicate the 95% confidence interval.

Scanner	Aperio	Grundium	Hamamatsu	Philips	Average
**Aperio**	-	0.97 (0.82, 1.00)	0.97 (0.83, 1.00)	0.93 (0.79, 1.00)	0.96 (0.87, 0.99)
**Grundium**	0.97 (0.82, 1.00)	-	0.93 (0.77, 1.00)	0.97 (0.84, 1.00)	0.95 (0.87, 0.99)
**Hamamatsu**	0.97 (0.83, 1.00)	0.93 (0.77, 1.00)	-	0.90 (0.74, 0.97)	0.93 (0.81, 0.99)
**Philips**	0.93 (0.79, 1.00)	0.97 (0.84, 1.00)	0.90 (0.74, 0.97)	-	0.93 (0.80, 0.99)

Table A8. Analysis of borderline cases comparing the prevalence of borderline cribriform morphology, as annotated by two experienced uropathologists, between true negative and false positive predictions. Type indicates the cohort's validation status.

		**True Negatives**			**False Positives**			
**Cohort**	**Type**	n	Borderline	%	n	Borderline	%	*p-value**
STHLM3	Internal	452	22	5%	24	10	42%	**¡0.001**
SUH	Internal	26	1	4%	5	2	40%	0.06
MUL	External	55	7	13%	27	9	33%	**0.038**
SCH	External	43	7	16%	2	2	100%	**0.036**
Overall	Internal	478	23	5%	29	12	41%	**¡0.001**
Overall	External	98	14	14%	29	11	38%	**0.008**

* Fisher's exact test

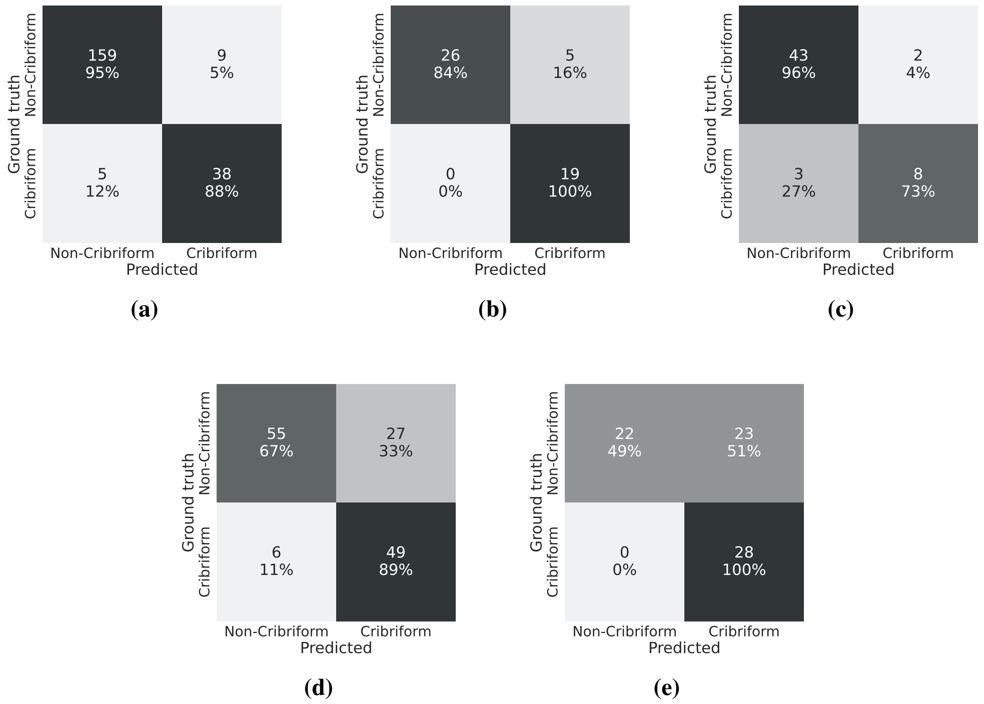

Figure A1. Confusion matrices on predictions for cohorts (a) STHLM3 (b) SUH (c) SCH (d) MUL (e) AMU

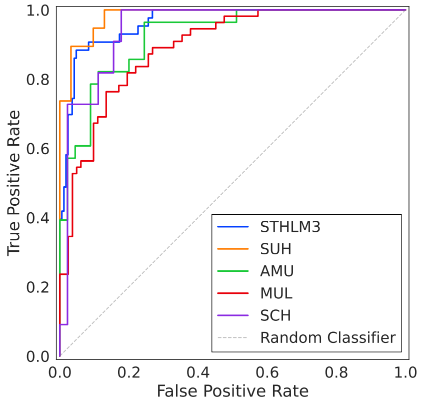

Figure A2. Receiver operating characteristic curves showing model performance for the different cohorts.

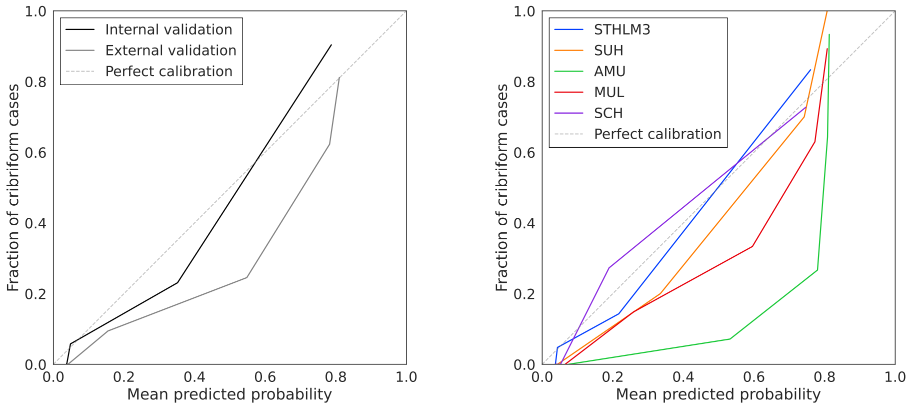

(a) **(b)**

Figure A3. (a) Calibration curves demonstrating the relationship between predicted probabilities and observed frequencies of cribriform morphology. (b) Calibration curves demonstrating the relationship between predicted probabilities and observed frequencies of cribriform morphology for the different cohorts.

Table A9. Compliance with STARD-AI reporting standards for AI-based diagnostic accuracy research. An item-by-item checklist, indicating the manuscript page or section where each criterion is addressed. Each item is evaluated as “Satisfied”, “Partially satisfied”, or “Not applicable”.

No.	STARD-AI Item	Location (Page/Section)	Context / Evidence	Status
1	Identification as AI diagnostic accuracy study in title/abstract	Title; Abstract	Title includes AI-based detection; Abstract reports study design.	Satisfied
2	Structured abstract with design, methods, results, conclusions	Abstract	Abstract structured into Background, Methods, Results, and Conclusion with clear diagnostic accuracy metrics.	Satisfied
3	Scientific and clinical background; intended use of index test	Introduction; Discussion	Describes clinical importance of cribriform morphology, underreporting, interobserver variability, and role of AI as decision support.	Satisfied
4	Study objectives and hypotheses	End of Introduction	Aim to develop and validate an AI model for cribriform morphology detection.	Satisfied
5	Prospective or retrospective design	Methods – Study design	Explicitly described as a retrospective, multi-cohort study.	Satisfied
6	Ethics approval or waiver	Ethical considerations	Ethical approvals and waivers detailed for all participating cohorts.	Satisfied
7	Inclusion and exclusion criteria	Methods – Data and participants; Protocol	Inclusion restricted to prostate core needle biopsies; detailed criteria referenced to published protocol.	Satisfied
8	Basis for identifying eligible participants	Methods – Data and participants; Protocol	Eligibility based on availability of prostate biopsies.	Satisfied
9	Setting, location, and dates	Methods – Data and participants; Ethical considerations; Protocol	International multi-centre cohorts with collection dates specified.	Satisfied
10	Consecutive, random, or convenience series	Methods – Data and participants; Protocol	Cohort-based random sampling described; exact sampling strategy detailed in protocol.	Satisfied
11	Source of data and purpose of collection	Methods – Data and participants	Routine diagnostic data reused for AI development and validation.	Satisfied
12	Who annotated the data and their expertise	Methods – Outcome	Annotations provided by expert uropathologists.	Satisfied
13	Devices and software used	Methods – Data and participants; Model development	Scanner vendors listed; deep learning architecture and software described.	Satisfied
14	Data acquisition and pre-processing protocols	Methods – Model development	Patch extraction, resolution, tissue segmentation, and preprocessing steps reported.	Satisfied
15a	Index test description sufficient for replication	Methods – Model development; Supplement Section B	Model architecture, MIL framework, augmentation, ensemble strategy described.	Satisfied
15b	Development, training, and validation datasets	Methods – Data and participants; Abstract	Training, internal validation, and external validation datasets described.	Satisfied
15c	Definition of cut-offs for test positivity	Methods – Statistical analysis	Binary operating point fixed at probability threshold of 0.5.	Satisfied
15d	Intended end-users and required expertise	Introduction; Discussion	Model positioned as decision-support for pathologists.	Satisfied
16a	Reference standard details for replication	Methods – Outcome	Cribriform morphology defined using ISUP 2021 consensus criteria; annotation procedures detailed.	Satisfied
16b	Rationale for reference standard	Methods – Outcome; Introduction	ISUP consensus morphology described as accepted clinical standard.	Satisfied
16c	Cut-offs used in the reference standard	Methods – Outcome	Binary slide-level presence/absence of cribriform morphology.	Satisfied

No.	STARD-AI Item	Location	Context / Evidence	Status
17a	Access to clinical information or reference results by index test	Methods – Model development	Model trained and inferred using WSIs only, without clinical metadata.	Satisfied
17b	Access to index test results by reference standard assessors	Methods – Outcome	Pathologists annotated slides independently of model outputs.	Satisfied
18	Statistical methods for diagnostic accuracy	Methods – Statistical analysis	AUC, sensitivity, specificity, Cohen's kappa, bootstrapped confidence intervals reported.	Satisfied
19	Handling of indeterminate results	Methods – Outcome; Statistical analysis	Borderline cases defined and analysed separately; classified as negative in main analyses.	Satisfied
20	Handling of missing data	Results – Dataset characteristics	No missing slide-level outcome data reported.	Satisfied
21	Analyses of variability	Methods – Statistical analysis; Results	Inter-rater variability, cross-scanner reproducibility, and cohort-level analyses performed.	Satisfied
22	Intended sample size and determination	Methods – Statistical analysis; Protocol reference	Sample size driven by available cohorts; prespecified in published protocol.	Satisfied
23	Algorithmic bias and fairness assessment	Discussion	International multi-cohort validation discussed; limitations and generalisability addressed.	Satisfied
24	Participant flow diagram	N/A	Study using available data from cohorts, no inclusion/exclusion was set.	Not applicable
25	Baseline demographic and technical characteristics	Results – Table 1	Age, PSA,Gleason score, ISUP grade reported.	Satisfied
26a	Distribution of disease severity	Results – Table 1; Table A8	Cribriform, borderline, and non-cribriform cases frequency reported.	Satisfied
26b	Distribution of alternative diagnoses	Methods – Outcome; Table 1	Gleason score and ISUP grade distributions reported across cohorts.	Satisfied
27	Interval and interventions between tests	Methods – Study design	Retrospective design; AI and pathologists assessed same digitised slides.	Satisfied
28	Representativeness of intended-use population	Discussion	Multi-national cohorts discussed in relation to intended pathology practice.	Satisfied
29	Differences between training and validation datasets	Methods – Data and participants; Results	Internal vs external cohorts, scanners, and laboratories explicitly separated.	Satisfied
30	Cross-tabulation of index and reference results	Results – Figure 2; Figure A1	Confusion matrices presented for internal and external validation as well as all cohorts.	Satisfied
31	Estimates of diagnostic accuracy and precision	Results – Tables 2; Figures 2–3; Figure A2	ROC curves, AUC, kappa, sensitivity, specificity with 95% confidence intervals reported.	Satisfied
32	Adverse events from testing	N/A	Non-invasive, retrospective diagnostic accuracy study.	Not applicable
33	Study limitations, bias, uncertainty, and generalisability	Discussion	Calibration drift, interobserver variability, and external performance limitations discussed.	Satisfied
34	Implications for practice and intended use	Discussion; Conclusion	Potential role in supporting pathologists and improving diagnostic consistency described.	Satisfied
35	Ethical issues and fairness of AI use	Ethical considerations; Discussion	Ethical approvals, data anonymisation, and responsible AI use discussed.	Satisfied
36	Registration number and registry	Methods; Protocol	Reference to published, prespecified study protocol.	Satisfied
37	Access to full study protocol	Methods;	Publicly available protocol cited.	Satisfied

No.	STARD-AI Item	Location	Context / Evidence	Status
38	Funding sources and roles	Acknowledgements	Funding sources disclosed; funders had no role in analysis or reporting.	Satisfied
39	Commercial interests	Conflict of interest statement	Shareholdings disclosed; no undisclosed competing interests.	Satisfied
40a	Availability of datasets and code	Data and Code Availability Statement	Data and code not publicly available due to privacy and ethical constraints.	Satisfied
40b	Auditability and stored outputs	Data and Code Availability Statement	Raw model predictions available upon reasonable request.	Satisfied

B Materials and Methods

B.1 Data Preparation

For STHLM3 and STG, pixel-wise annotations were made. The lead pathologist created pixel-wise annotations (marking cribriform regions) on only one digital version of each slide. Due to differences in how each scanner positioned the slide during digitisation, these annotations could not be directly transferred to other digital versions of the same slide. To address this limitation and increase our training data, we designed a simple phase correlation-based image registration algorithm. For slides with multiple scans, we created binary tissue segmentation masks and used a Fast Fourier Transform-based cross-correlation algorithm to align annotations across different digital versions of the same physical glass slide.

B.2 Model Development

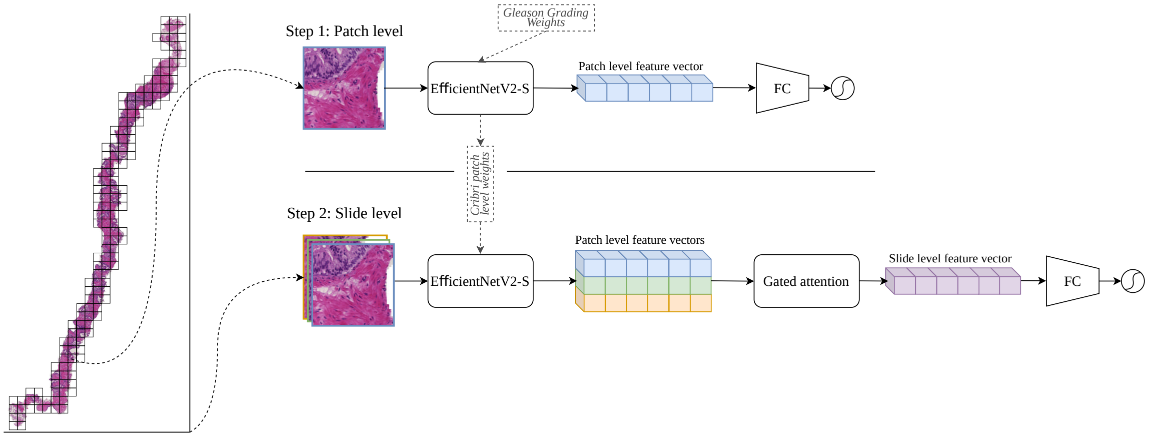

Figure A4. Model architecture illustrating the patch level and slide level classifiers for cribriform morphology detection in prostate cancer.
Definition of abbreviations: FC = Fully connected layer.

We implemented a two-step transfer learning procedure to enhance performance, convergence speed, and generalisation. The model architecture and transfer learning process are illustrated in Figure A4. This approach furthermore enabled the incorporation of SUH cohort data during Step 2, as this dataset lacked pixel-level annotations required for fully supervised training.

Step 1: Fully Supervised Patch Level Classifier. We created a patch level classifier using a convolutional neural network. EfficientNetV2-S was chosen as the backbone due to its high performance and relatively low resource usage [1]. To detect cribriform morphology at the patch level, we fine-tuned an EfficientNetV2-S encoder, which was previously part of a multiple instance learning (MIL) model trained on a large Gleason grading dataset [2]. The feature vector was passed through a fully connected layer with a sigmoid activation function, producing a probability score for cribriform morphology.

Step 2: Weakly Supervised Slide Level Classifier. We then developed a slide level classifier by transferring the cribriform patch level encoder weights into a MIL architecture. The encoder weights were not frozen, allowing for further fine-tuning during slide-level training. Bags of patches from a slide were processed through the encoder to create patch level feature vectors. These vectors were pooled together using gated attention to form a single slide level feature vector, which was then passed through a sequence of fully

connected layers with normalisation, activation, and dropout, followed by a sigmoid activation function to produce a slide level probability score.

B.3 Training

We used a binary cross-entropy loss function, weighted by the frequency of positive labels in the training data, with a static probability threshold of 0.5 for classification. The AdamW optimiser was employed with a one cycle learning rate scheduler for the patch level model (starting at $1 \cdot 10^{-5}$, peaking at $1 \cdot 10^{-4}$ after 1 epoch, and finally decreasing to $1 \cdot 10^{-6}$) and a constant learning rate of $3 \cdot 10^{-5}$ for the slide level model. The patch level classifier uses a weight decay of $1 \cdot 10^{-2}$ while the slide level model uses $1 \cdot 10^{-5}$. Data augmentations included random cropping, vertical and horizontal flips, colour and brightness jitter, sharpening, blurring, noise, JPEG compression, and random greyscale conversion. Furthermore, during training, for slides scanned multiple times on different scanners, we randomised which digital scan of a biopsy slide to use on each epoch. Hyperparameters are summarised in Tables A2 and A3. The models were trained for 8 and 32 epochs for the patch level and slide level respectively, with checkpoints every epoch, retaining only the checkpoint with the highest non-weighted Cohen's kappa on the hold-out fold. We used 10-fold cross-validation to evaluate the model during development. To avoid data leakage when transferring the Gleason grading weights, we employed protocol-defined splits. Platt scaling was applied by fitting a logistic regression model to the held-out folds in the training cross-validation.

For the patch level classifier, patches were labelled as cribriform-positive if they contained more than 2% of positively annotated tissue. Due to the constraints of needing pixel-wise annotations for the patch level classifier, the classifier could only be trained on data from the STHLM3 and STG cohorts. For the slide level classifier, bags of patches were labelled based on slide level annotations, enabling the classifier to be trained on the SUH cohort as well. To further utilise pixel-wise annotations in the STHLM3 and STG data, bags of patches from STHLM3 and STG were labelled as positive only if they contained a patch with cribriform morphology. For further regularisation, with the 50% overlap that the patches were extracted with, we could construct two sets of non-overlapping patches per WSI. For each forward pass of the model, one of these two non-overlapping sets was used. During validation, we used all extracted patches from a slide.

B.4 Inference

Our final model utilised a 10-fold ensemble approach derived from the cross-validation folds during model development. For inference, we extracted patches of size 256 by 256 pixels at a resolution of 1 μm per pixel, with 50% overlap between adjacent patches both vertically and horizontally. Patches were excluded if tissue content comprised less than 10% of the image. All extracted patches were passed through the model. To enhance prediction robustness, we applied test time augmentation with 5 iterations per ensemble model, using non-destructive transformations at a patch level, such as flipping and rotation. The final prediction was generated through soft voting, averaging predictions across all test time augmentation iterations and ensemble models.

B.5 Software and Hardware

Models and statistical analyses were implemented in Python 3.10 using Pytorch version 2.4 and Pytorch Lightning version 2.3. Support packages included Albumentations (1.4.12), LMDB (1.5.1), Numpy (2.1.3), Polars (1.14.0), Scipy (1.14.1), Scikit-learn (1.5.2), Timm (1.0.11). Plots were made using Matplotlib (3.9.2) and Seaborn (0.13.2). Models were trained using a single NVIDIA A100 80gb Tensor Core GPU. Inference was run on a single NVIDIA A100 40gb Tensor Core GPU. The extracted patches were encoded as JPEGs and saved to the Lightning Memory-Mapped Database (LMDB) format, as this allowed for efficient random reads which were needed in both phases of training. The resulting patch level and slide level models had

20.2 million and 21.9 million trainable parameters, respectively. The final training took 10 hours per data fold for a total of 100 GPU hours.

C Results

C.1 Cross-scanner Reproducibility

When examining cross-scanner reproducibility (Table A7), we utilised 71 slides from the STHLM3 internal validation set that had all been scanned on scanners from 4 different vendors. The subset included 19 slides that contained the cribriform pattern. The average pairwise Cohen's kappa values across scanners demonstrated high consistency, with Aperio achieving the highest average agreement at 0.96 (95% CI: 0.87, 0.99), followed by Grundium at 0.95 (95% CI: 0.87, 0.99), while Hamamatsu and Philips showed average Cohen's kappa values of 0.93 (95% CI: 0.78, 0.99) and 0.93 (95% CI: 0.81, 0.99) respectively. The highest level of agreement was observed between Aperio and Grundium scanners (Cohen's kappa: 0.97, 95% CI: 0.80, 1.00) and between Aperio and Hamamatsu scanners (Cohen's kappa: 0.97, 95% CI: 0.82, 1.00). The lowest agreement was found between Hamamatsu and Philips scanners with a Cohen's kappa value of 0.90 (95% CI: 0.73, 0.97).

References

[1] Tan M and Le QV. EfficientNetV2: Smaller Models and Faster Training. arXiv [Preprint] 2021. Available from: `http://arxiv.org/abs/2104.00298`

[2] Mulliqi N, Blilie A, Ji X, Szolnoky K, Olsson H, Boman SE, et al. Foundation Models – A Panacea for Artificial Intelligence in Pathology? arXiv [Preprint] 2025. Available from: `http://arxiv.org/abs/2502.21264`
